# Aging-Related Metabolome Analysis of the Masseter Muscle in Senescence-Accelerated Mouse-Prone 8

**DOI:** 10.3390/ijms25179684

**Published:** 2024-09-07

**Authors:** Yoshiaki Kato, Teruhide Hoshino, Yudai Ogawa, Keisuke Sugahara, Akira Katakura

**Affiliations:** 1 Department of Oral Pathobiological Science and Surgery, Tokyo Dental College, Tokyo 101-0061, Japan; katouyoshiaki@tdc.ac.jp (Y.K.); ksugahara@tdc.ac.jp (K.S.); katakura@tdc.ac.jp (A.K.); 2 Department of Histology and Developmental Biology, Tokyo Dental College, Tokyo 101-0061, Japan; yogawa.asty519@gmail.com

**Keywords:** metabolites, metabolome analysis, masseter muscle, aging, senescence-accelerated mouse

## Abstract

Frailty is a vulnerable state that marks the transition to long-term care for older people. Early detection and prevention of sarcopenia, the main symptom of frailty, are important to ensure an excellent quality of life for older people. Recently, the relationship between frailty, sarcopenia, and oral function has been attracting attention. This study aimed to clarify the changes in metabolites and metabolic pathways due to aging in the masseter muscle of senescence-accelerated mouse-prone 8 (SAMP8) mice. A capillary electrophoresis-mass spectrometry metabolome analysis was performed on the masseter muscle of 12-week-old, 40-week-old, and 55-week-old mice. The expression of enzymes involved in metabolome pathways considered to be related to aging was confirmed using reverse transcription polymerase chain reaction. Clear metabolic fluctuations were observed between 12, 40-week-old, and 55-week-old SAMP8 mice. The extracted metabolic pathways were the glycolysis, polyamine metabolome, and purine metabolome pathways. Nine fluctuated metabolites were common among the groups. Spermidine and Val were increased, which was regarded as a characteristic change in the masseter muscle due to aging. In conclusion, the age-related metabolic pathways in SAMP8 mice were the glycolysis, polyamine metabolome, and purine metabolome pathways. The increased spermidine and Val levels in the masseter muscle compared with the lower limbs are characteristic changes.

## 1. Introduction

Frailty is a vulnerable state that marks the transition to long-term care for older people and an increased risk of health problems, fall incidents, and death [1]. Early detection and prevention of sarcopenia, the main symptom of frailty, are important to ensure an excellent quality of life for older people. Recently, the relationship between frailty, sarcopenia, and oral function has been attracting attention in research. For instance, in an epidemiological study, Murakami et al. conducted a survey of community-dwelling older people in Japan and reported an association between sarcopenia and masticatory function [2]. Iwasaki et al. reported that a lower maximum bite force was associated with an increased risk of frailty [3]. Moreover, the concept of oral frailty for the early detection of slight deterioration in older people was proposed, and the characteristic of poor diadochokinesis constituted the first step toward oral dysfunction. Oral frailty was also shown to be associated with the prognosis of curtailed longevity [4].

We have noted that there are only a limited number of defining studies that link the decline in oral health to aging in fundamental research, with the majority of studies focusing on the aging processes of animals’ limbs. Snow et al. measured the cross-sectional area of the soleus muscle in Fischer 344 Brown Norway (FBNF1) rats and reported the atrophy of fast-twitch muscles and notable changes in old age [5]. Guo et al. observed morphological and immunohistochemical changes in the gastrocnemius muscle of senescence-accelerated mouse-prone 8 (SAMP8) mice over time and reported that sarcopenia appeared when they were 40 weeks old [6]. Additionally, by conducting a metabolome analysis on the extensor digitorum longus (EDL) muscle of SAMP8 mice to identify metabolites that fluctuated due to aging, we observed and confirmed two aging-related metabolic changes in the EDL muscle of these mice [7]. Furthermore, Uchitomi et al. performed a metabolome analysis on the gastrocnemius muscle of aged C57BL/6J mice to identify metabolites that fluctuated because of aging [8]. They reported a decrease in glucose metabolites and polyamine metabolites, as well as an increase in neurotransmitters.

The masseter muscle plays a central role in mastication among the masticatory muscles, and its changes due to aging have been investigated in clinical studies [9,10]. We reported that masseter muscle atrophy in SAMP8 mice occurred at 40 weeks of age and that there were subsequent changes in muscle contraction characteristics [11]. However, there are no reports of the molecular biological examination of the masseter muscle during aging. Given this context, we focused on the metabolites in this study. While previous studies have suggested metabolic changes in major metabolic pathways, such as the glycolytic system, related details remain unclear. By clarifying the specific metabolic changes in the masseter muscle, we sought to contribute to the maintenance of masticatory function. The current study aimed to clarify the changes in metabolites and metabolic pathways in the masseter muscle due to aging.

## 2. Results

### 2.1. Body Weight

**Figure 1:**
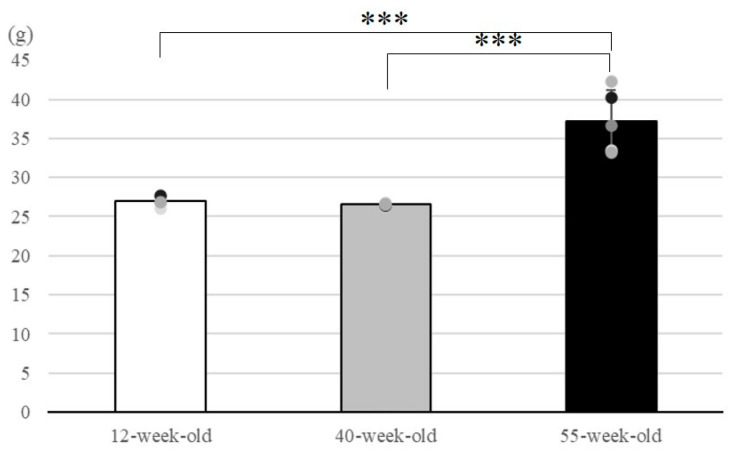
Body weights of SAMP8 mice at each age were measured when the masseter muscle was extracted. Body weight at 55 weeks showed a significant increase compared to that at 12 and 40 weeks. *** *p* < 0.001. *X*-axis: each age; *Y*-axis: body weight (g). Plotting points show the weight of each mouse at each stage. The error bars represent the standard deviation.

The mean body weight was 27.00 ± 0.67 g for 12-week-old, 26.58 ± 0.08 g for 40-week-old, and 37.16 ± 4.05 g for 55-week-old mice (Figure 1). The body weight increased between 12 and 55-week-old and between 40 and 55-week-old mice; however, no difference in weight was found between 12-week-old and 40-week-old mice.

### 2.2. Metabolome Analysis

**Figure 2:**
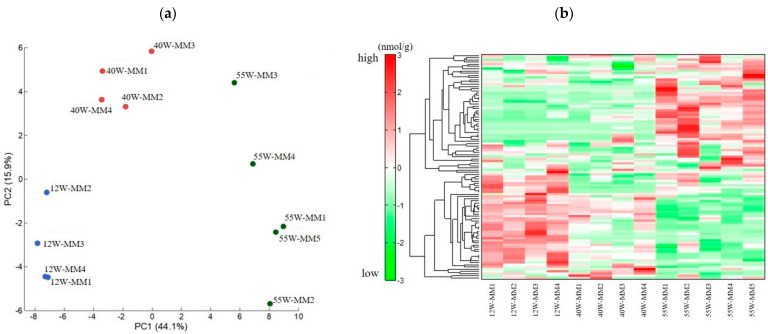
(**a**) Principal component analysis (PCA) of metabolomic datasets of the masseter muscle (MM) at 12, 40, and 55 weeks. Plots of 12−week−old (blue), 40−week−old (red), and 55−week−old (green) mice are clearly distinguished on the first principal component axis (*X*−axis). (**b**) Hierarchical cluster analysis (HCA) of metabolite changes at 12, 40, and 55 weeks. The horizontal axis shows the sample names corresponding to the samples used in Figure 2a (12W−MM1 to 12W−MM4 for 12−week−old mice, 40W−MM1 to 40W−MM4 for 40−week−old mice, and 55W−MM1 to 55W−MM5 for 55−week−old mice). Red indicates that the relative content of metabolites is high, whereas green indicates that the relative content of metabolites is low.

A principal component analysis (PCA) showed that the metabolites contributing to the first principal component could be clearly distinguished at each age (Figure 2a). Hierarchical cluster analysis (HCA) also revealed changes in metabolite profiles (Figure 2b). Moreover, clear metabolic fluctuations were observed between the 12 and 55-week-old, as well as the 40 and 55-week-old mice.

#### 2.2.1. Change in Metabolite Levels between 12 and 40−Week−Old Mice

Out of 30 metabolites that exhibited fluctuations (Table S1), 2 were characterized by elevated levels, whereas 28 showed decreased levels. Moreover, significant changes in the purine metabolism and glycolysis pathways were observed.

#### 2.2.2. Change in Metabolite Levels between 12 and 55−Week−Old Mice

A total of 51 metabolites exhibited fluctuations between 12-week-old and 55-week-old (Table S2) mice. Out of these metabolites, 17 showed elevated levels, whereas 34 displayed decreased levels. In particular, decreased levels of carnosine, β-Ala, and His, which are involved in muscle aging, were observed.

#### 2.2.3. Changes in Metabolite Levels between 40 and 55−Week−Old Mice

**Figure 3:**
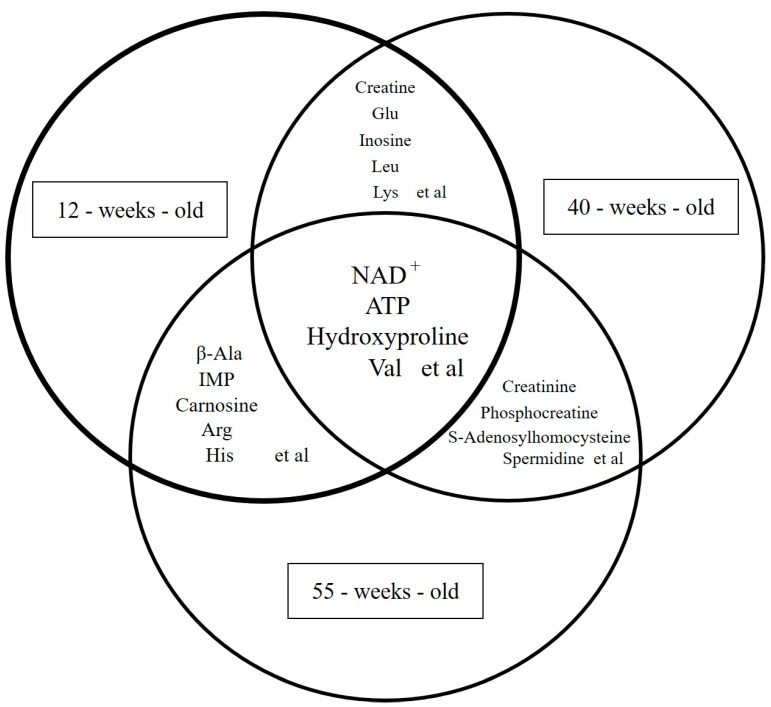
Venn diagram of metabolites that fluctuated in all groups. This analysis focused on 116 central metabolites related to central energy metabolic pathways. The fluctuated metabolites common among the groups were Gly, NAD^+^, urea, 2-phosphoglyceric acid, 3-phosphoglyceric acid, phosphoenolpyruvic acid, Val, hydroxyproline, and ATP.

Out of 36 metabolites that exhibited significant differences (Table S3), 20 showed elevated levels, whereas 16 displayed decreased levels. Significant changes in the polyamine metabolism, purine metabolism, and glycolysis pathways were noted. Gly, NAD^+^, urea, 2-phosphoglyceric acid, 3-phosphoglyceric acid, phosphoenolpyruvic acid, Val, hydroxyproline, and ATP were identified as the metabolites that exhibited fluctuations and were common among the groups (Figure 3). All of these metabolites decreased with aging, except for Val, which increased between 40 weeks and 55 weeks.

**Figure 4:**
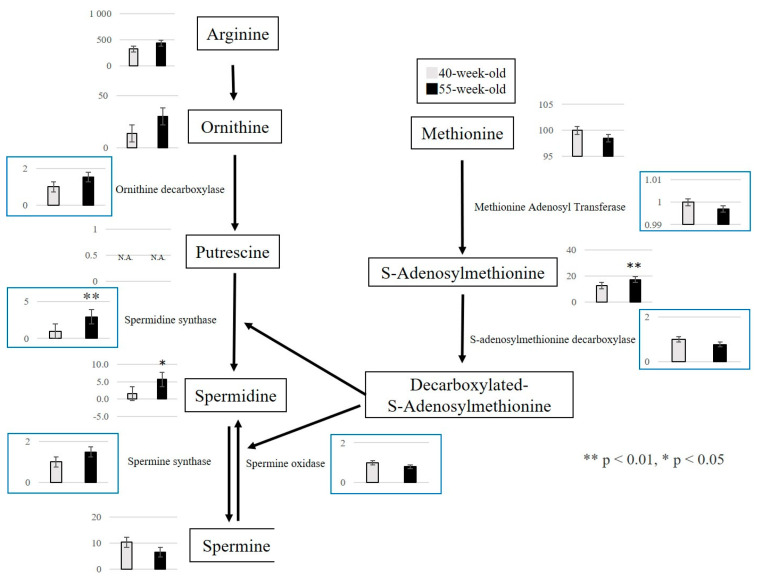
Pathway map of polyamine metabolism. Metabolite levels in polyamine metabolism in 40−week−old and 55−week−old mice. ** *p* < 0.01, * *p* < 0.05. Gene expressions of involved enzymes are enclosed in the blue squares. *X*-axis: each age; *Y*-axis: relative mRNA levels. N.A.: Not Available.

**Figure 5:**
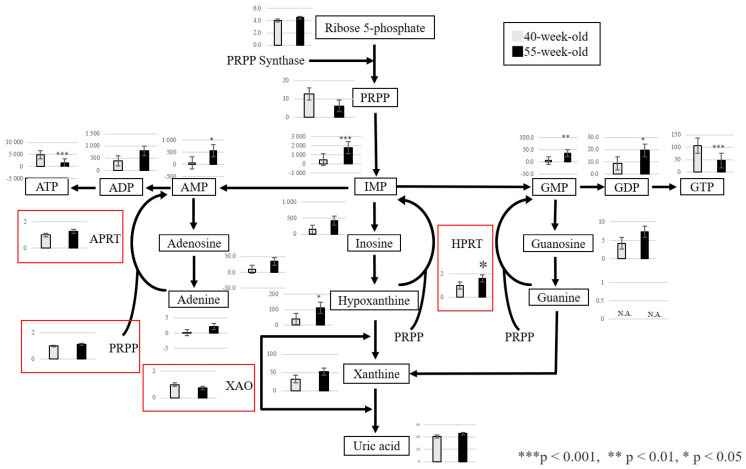
Pathway map of purine metabolism. Metabolite levels in purine metabolism in 40−week−old and 55−week−old mice. *** *p* < 0.001, ** *p* < 0.01, * *p* < 0.05. Gene expressions of involved enzymes are enclosed in red squares. *X*−axis: each age; *Y*−axis: relative mRNA levels. N.A.: Not Available.

**Figure 6:**
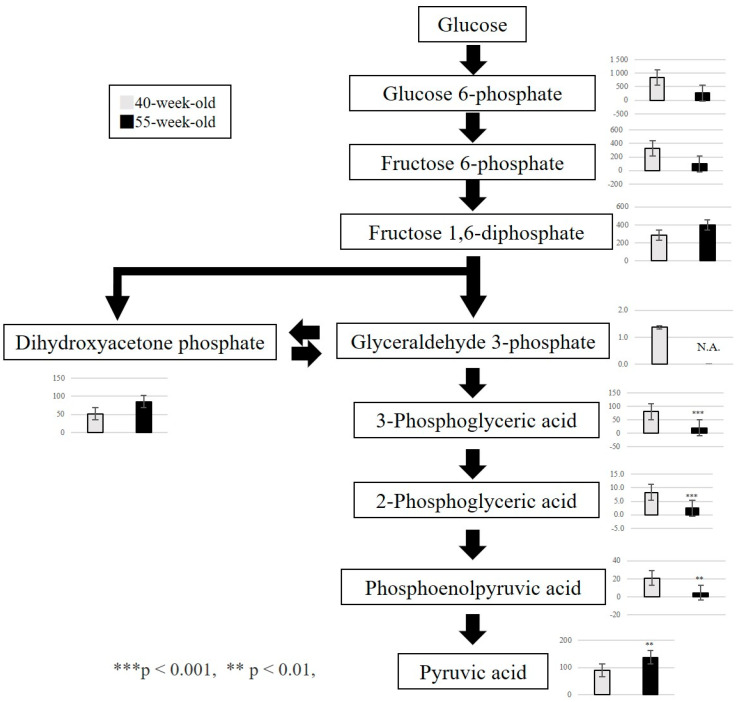
Pathway map of glycolysis. Metabolic changes related to polyamine metabolism in 40−week−old and 55−week−old mice. *** *p* < 0.001, ** *p* < 0.01. N.A.: Not Available.

From the above results, we focused on the metabolites that exhibited fluctuations between 40 and 55 weeks. We confirmed that three metabolic pathways were primarily affected by the aging process. First, the polyamine metabolism pathway was associated with cell proliferation, protein synthesis, and nucleic acid synthesis. A significant increase in S-adenosylmethionine and spermidine was observed (Figure 4). Second, the purine metabolism pathway was related to purine nucleotide metabolism and ATP resynthesis. In this regard, a significant increase in IMP, GMP, GDP, and hypoxanthine and a significant decrease in GTP and ATP were detected (Figure 5). Third, we observed a decrease in 3-phosphoglyceric acid, 2-phosphoglyceric acid, and phosphoenolpyruvic acid, which are engaged in glycolysis (Figure 6). Additionally, we observed an increase in choline, which is an essential nutrient produced in the liver, and a decrease in phosphocreatine, which is used for the synthesis of ATP and the production of creatinine (a reaction product).

### 2.3. Quantitative Analysis of Metabolites Using RT-PCR

This study focused on the polyamine and purine metabolism pathways that fluctuated between 40 and 55-week-old mice because metabolite levels showed a change in 55-week-old mice compared to 40-week-old mice. For target genes in the polyamine metabolism pathway, we confirmed the expression of enzymes involved in the polyamine and purine metabolism pathways between 40-week-old and 55-week-old mice. In the polyamine metabolism pathway, the expression of the spermidine synthase gene (Srm) significantly increased in 55-week-old mice compared with 40-week-old mice (*p* = 0.002) (Figure 4). In the purine metabolism pathway, the expression of the hypoxanthine phosphoribosyl transferase gene (HPRT), an enzyme used in the salvage circuit of ATP resynthesis (*p* = 0.048), also significantly increased (Figure 5).

## 3. Discussion

Our results suggested the occurrence of changes in the metabolites and metabolic pathways of the masseter muscle were due to aging and indicated that the age-related metabolic pathways of the masseter muscle in SAMP8 mice were likely to be the glycolysis, polyamine metabolome, and purine metabolome pathways. In particular, spermidine levels were increased in the masseter muscle compared to the hind limbs, which was considered to be a characteristic change in the masseter muscle.

The body weight of SAMP8 mice is said to increase between 8 and 24 weeks of age [12]. Similarly, in this study, the body weight tended to increase as the mice aged, and there was no clear individual difference during the raising stage.

Aging physiologically reduces food intake, and anorexia due to aging leads to a lack of energy, malnutrition, and weight loss [13]. We measured the amount of food intake at 55 weeks for five mice over time (Figure S1) and observed that the amount of food intake at 55 weeks significantly decreased compared with those at 12 and 40 weeks. However, because this was not accompanied by weight loss, we inferred that this was not a case of simple undernutrition. Consequently, we think that it is necessary to evaluate other factors, such as lean body mass and whole-body muscle mass, to clarify the relationship between body weight and food intake and also to measure nutritional indicators, such as blood cholesterol and hemoglobin levels.

The masseter muscle predominantly consists of fast-twitch muscle fibers in mice, with many MyHC type II fibers distributed among the muscle fibers [14]. Fast-twitch muscle fibers have high enzymatic activity and creatine kinase activity that contribute to glycolysis and have excellent anaerobic metabolic capacity [15]. Studies indicate that glycolytic metabolites in skeletal muscle decrease with aging [5]. In the gastrocnemius muscle, fructose 1,6-diphosphate, dihydroxyacetone phosphate, and glyceraldehyde 3-phosphate (upstream metabolites) decreased [8]. Our results indicated a decrease in glycolytic metabolites, namely, 3-phosphoglyceric acid, 2-phosphoglyceric acid, and phosphoenolpyruvic acid (downstream metabolites). MyHC type IIb fibers decrease in the masseter muscle of SAMP8 mice, notably between 40- and 55-week-old mice [9]; this decline could be associated with the aforementioned decrease. The dephosphorylation of 1,3-bisphosphoglyceric acid produces 3-phosphoglyceric acid. This reaction is accompanied by ATP production through the phosphorylation of ADP. Phosphoenolpyruvic acid is produced by the dehydration of 2-phosphoglyceric acid. This reaction is considered to produce pyruvic acid simultaneously with ATP by the transfer of a phosphate group to ADP. We suggest that the decrease in 3-phosphoglyceric acid, 2-phosphoglyceric acid, and phosphoenolpyruvic acid in the masseter muscle is caused by the consumption of a large amount of ATP required for masticatory movements.

Polyamines are substances present in the cells of many organisms and are indispensable for cell proliferation and differentiation. It also plays an important role in cellular function [16]. Polyamines have many amino groups, and spermidine and spermine are typical. Proceeding with the analysis, spermidine is an autoinducer associated with autophagy [17], which is attracting attention not only as a disease but also as a factor that controls aging [18]. Spermidine concentrations have been reported to decrease with age [19]. Moreover, its concentration decreases significantly in the gastrocnemius muscle of C57/BL6J mice [8] and the EDL muscle of SAMP8 mice [7]. In this study, we observed a significant increase in spermidine levels in the masseter muscle. Furthermore, the expression of Srm in the masseter muscle was greater between 40-week-old and 55-week-old mice, as shown by reverse transcription polymerase chain reaction (RT-PCR). We believe that it participated in the synthesis of spermidine in the polyamine metabolism pathway. The masseter muscle’s involvement in the masticatory function is essential to maintain a normal life. Additionally, existing reports have proven that autophagy is activated in the masseter muscle compared with the hind limbs [20] and that the masseter muscle in both young and older adults shows similar performance to that in rats [21]. Additionally, carnosine, a dipeptide consisting of β-Ala and His, is abundant in the muscles and nervous tissues. In this study, carnosine, β-Ala, and His decreased between 12 and 55 weeks. Based on these findings, we think that the masseter muscle, which is used more frequently than the hind limbs, is more susceptible to fatigue and aging, and a compensatory increase in spermidine induces autophagy activation, which may be correlated with maintaining muscle function. Hence, evaluating the autophagy function in aging muscle tissues is important.

S-adenosylmethionine is involved in DNA methylation and is reported to increase with aging [22,23,24,25]. DNA methylation involves adding a methyl group to cytosine provided by SAM through the action of DNA methyltransferase, converting it into methylcytosine. Furthermore, DNA methylation is a sign of slight changes in tissue composition, such as tissue inflammation and fibrosis [26], and has become the subject of several studies as an epigenetic biomarker of aging [27,28]. In this study, we confirmed that the levels of SAM increased in SAMP8 mice from 12-week-old to 55-week-old and 40-week-old to 55-week-old. We believe that this represents a significant metabolic change associated with age in the masseter muscle.

The purine metabolism pathway is engaged in ATP resynthesis during movement. Phosphocreatine is required for ATP synthesis [29], and hypoxanthine and IMP increase with movement [30]. This study confirmed the decrease of phosphocreatine as a contributing factor to the decrease in ATP. It also provided evidence that the increase in hypoxanthine and IMP was associated with the masticatory function of the masseter muscle in SAMP8 mice. We confirmed that the increased demand for energy sources, such as ATP and GTP, was observed in both the purine metabolism pathway and glucose metabolism. Furthermore, the expression of HPRT was confirmed by RT-PCR. We suggest that the salvage pathway for resynthesis was activated, which may have led to the increase in HPRT. This may indicate that the activation of the salvage circuit provides ATP and contributes to maintaining the functions of the masseter muscle.

A total of nine fluctuated metabolites were found to be common among the groups, and all decreased with age; however, Val increased between 40 and 55 weeks. Val, an amino acid in the branched-chain amino acid (BCAA) metabolic pathway secreted by skeletal muscles, especially during exercise, acts on other tissues to increase energy expenditure. In contrast, in the BCAA metabolic pathway, Leu and Ile were significantly decreased between 12 and 40 weeks. These results suggest that the BCAA metabolic pathway may play a significant role in the aging of the masseter muscle in SAMP8 mice. Additionally, the fluctuation of Val metabolites in the masseter muscle with aging may also have an impact. We reported that there were metabolites that fluctuated with aging in the EDL muscle of SAMP8 mice [4]. Among these metabolites, Val and Leu decreased with age. The increase in Val may represent a specific change in the masseter muscle compared with the lower limbs.

All of these metabolites affect the aging process of the masseter muscle. Hydroxyproline is the main component of collagen proteins, in which proline residues are hydrolyzed and are biosynthesized by protein degradation. It is inferred that a significant decrease in hydroxyproline may decrease the degradation of collagen and the synthesis of proteins. Recently, it has been reported that oral function is associated with frailty and sarcopenia [2,3,4]. Additionally, sarcopenia is associated with the masticatory function [2]. These findings suggest that when sarcopenia is present, it will also have progressed to muscles involved in mastication, causing a reduction in masticatory function over time.

In the present study, we focused on the masseter muscle, which plays a central role in the masticatory function, and observed age-related metabolic changes in this muscle using SAMP8 mice. Metabolites occasionally suppress transcriptional expression and protein functions. We considered that investigating metabolites may clarify the mechanism of aging in the masseter muscle. The metabolic changes found in this study may be an indicator of exploring the molecular, biological, functional, and morphological changes in the masseter muscle during aging. Additionally, in the future, we believe that we may help in understanding the aging mechanism of the masseter muscle by evaluating gene expression, including microarray and bioinformatic analyses, after replacement therapy with ingestion of a polyamine-rich diet. Given that there have been reports on traditional Japanese Earsten Kampo medicine for frailty prevention [31], we would like to investigate what kind of changes are brought about in the masseter muscle of SAMP8 mice by this intervention.

A limitation of this study is that aging-related changes in mice may differ from those in humans. SAMP8 mice have a shorter lifespan than typical aged animals [32] and develop a sarcopenia phenotype [6]; we expect them to be used in future studies on aging. In our study, SAMP8 mice were used to understand the characteristics of aging in the masseter muscle. It was thought that by conducting a similar survey in wild-type mice, it would be possible to better understand the changes in the masseter muscle due to aging. Additionally, SAMR1 is usually used as a “control” for SAMP8 mice. To further clarify the phenotype of the masseter muscle during aging, we think that there is a need to compare it with younger mice as in previous studies [11]. In addition, there are problems associated with aging research on samples. Aging animals may die, making it difficult to secure a larger number of samples than expected. In this study, the number of samples was determined based on the literature [7,8]. These studies yielded clear results. Moreover, the mean life expectancy of the SAMP8 mice used in this study was approximately 40 weeks. Some mice died during breeding; therefore, we standardized sample sizes. The findings clearly differentiated between PCA and HCA. In the future, we aim to increase the sample size and verify the study results.

## 4. Materials and Methods

### 4.1. Animal Experiment

In this study, male SAMP8 mice (SAMP8/Ta Slc) were utilized as samples. SAMP8 mice were purchased from Sankyo Labo service corporation (Tokyo, Japan). SAMP8 mice are a type of mice whose age accelerates between 16 weeks and 20 weeks and are often used in aging research. The mice were raised for categorization into each of the following groups: 12-week-old (*N* = 4), a stage before the start of accelerated aging; 40-week-old (*N* = 4), the mean lifespan of SAMP8 mice; 55-week-old (*N* = 5), a stage that exceeded the mean lifespan. In this study, the same species and the same ages were used as in previous studies [9]. The mice were raised in a 125 × 213 × 125 mm aluminum cage and were freely given food (Lab MR-A1) and water. After administering general anesthesia with isoflurane, the mice were euthanized by dislocating the cervical spines, and the masseter muscle was extracted. The left side of the muscle was used for metabolome analysis, whereas the right side was utilized for RT-PCR.

The experiments were conducted in accordance with the National Institutes of Health Guide for the Care and Use of Laboratory Animals and were approved by the Tokyo Dental College Institutional Animal Care and Use Committee (approval number: 202601; approval date: 1 August 2020). This study was carried out in compliance with the ARRIVE guidelines.

### 4.2. Metabolite Extraction and Capillary Electrophoresis–Mass Spectrometry (CE-MS) Metabolome Analysis

Approximately 50 mg of frozen tissue was plunged into 750 µL of 50% acetonitrile/Milli-Q water containing internal standards (20 μM) (H33041002, Human Metabolome Technologies, Inc., Tsuruoka, Japan) at 0 °C to inactivate enzymes. The tissue was homogenized five times at 3500 rpm for 1 min using a tissue homogenizer (Micro Smash MS100R, Tomy Digital Biology Co., Ltd., Tokyo, Japan); the homogenate was then centrifuged at 2300× *g* and 4 °C for 5 min. Subsequently, 400 µL of the upper aqueous layer was centrifugally filtered through a Millipore 5 kDa cutoff filter at 9100× *g* at 4 °C for 120 min to remove proteins. The filtrate was concentrated in the form of a dried pellet by centrifugation and re-suspended in 50 µL of milli-Q water for CE-MS analysis. This analysis focused on 116 central metabolites related to central energy metabolic pathways.

Metabolome analysis was performed with the *C-SCOPE* package (ver.3.1.2: from Human Metabolome Technologies, Inc., Yamagata, Japan) using capillary electrophoresis time-of-flight mass spectrometry (CE-TOFMS) for cation analysis and CE-tandem mass spectrometry (CE-MS/MS) for anion analysis based on the methods described previously [33,34]. CE-TOFMS analysis was conducted using an Agilent CE capillary electrophoresis system equipped with an Agilent 6210 time-of-flight mass spectrometer (Agilent Technologies, Waldbronn, Germany). The spectrometer was scanned from *m*/*z* 50 to 1000 (1). Peaks were extracted using MasterHands (ver.2.17.1.11), automatic integration software (Keio University, Tsuruoka, Yamagata, Japan) (3), and MassHunter Quantitative Analysis B.04.00 (Agilent Technologies) in order to obtain peak information including *m/z*, peak area, and migration time (MT). Signal peaks were annotated according to the HMT metabolite database based on their *m*/*z* values with the MTs. HCA and PCA were performed using PeakStat (ver.3.18) and SampleStat (ver.3.14) software, respectively.

### 4.3. Quantitative Analysis of Gene Expression in the Masseter Muscle with Aging

Total RNA extraction of the masseter muscle was performed using the RNeasy Mini Kit (Qiagen, Hilden, Germany) and Proteinase K (Takara Bio, Shiga, Japan). The cDNA was prepared using the QuantiTect Reverse Transcription Kit (Thermo Fisher Scientific, Waltham, MA, USA). The Double Delta Ct Value (ΔΔCt) method was used for quantification, and the relative expression level was compared to the expression of housekeeping genes in each sample. For gene expression analysis, TaqMan^®^ gene expression assays (Thermo Fisher Scientific, Waltham, MA, USA) were employed in the RT-PCR 7500 system (Thermo Fisher Scientific). TaqMan^®^ probes (Thermo Fisher Scientific) used Methionine Adenosyl Transferase 2A (Mat2a) (Mm00728688_s1), spermidine synthase gene (Srm) (Mm00726089_s1), spermine synthase (Sms) (Mm00786246_s1), Ornithine decarboxylase1 (Odc1) (Mm02019269_g1), spermine oxidase (Smox) (Mm01275475_m1), and S-adenosylmethionine decarboxylase (Amd2) (Mm04207265_gH). For the purine metabolism pathway, TaqMan^®^ probes used HPRT (Mm03024075_m1), phosphoribosyl pyrophosphate 1 (Prps1) (Mm00727494_s1), adenine phosphoribosyl transferase (Aprt) (Mm04207855_g1), and xanthine dehydrogenase (Xdh) (Mm0044). In addition, the housekeeping gene β-actin (Mm00607939_s1) was employed.

### 4.4. Statistical Analyses

Statistical analyses were performed with IBM SPSS Statistics version 26 (IBM, Armonk, NY, USA) software. Moreover, to obtain statistics related to body weight, Tukey’s test was performed for the multiple comparison test. The metabolites, RT-PCR of 40-week-old and 55-week-old mice, were compared using Welch’s t-test. The significance threshold was set at 5% (*p* < 0.05).

## 5. Conclusions

Our results suggest that age-related metabolic pathways of the masseter muscle in SAMP8 mice are likely related to the glycolysis, polyamine metabolome, and purine metabolome pathways. In particular, the increased levels of spermidine and Val in the masseter muscle compared with the lower limbs are considered to be characteristic changes in the masseter muscle.

## Data Availability

The datasets analyzed during the current study are not publicly available.

## Supplementary Materials

The following supporting information can be downloaded at https://www.mdpi.com/article/10.3390/ijms25179684/s1.
