# Supplementary figures and images for: Aging-Related Metabolome Analysis of the Masseter Muscle in Senescence-Accelerated Mouse-Prone 8
Source: Int J Mol Sci. 2024 Sep 7;25(17):9684. doi: 10.3390/ijms25179684 (PMC11395484; doi:10.3390/ijms25179684)

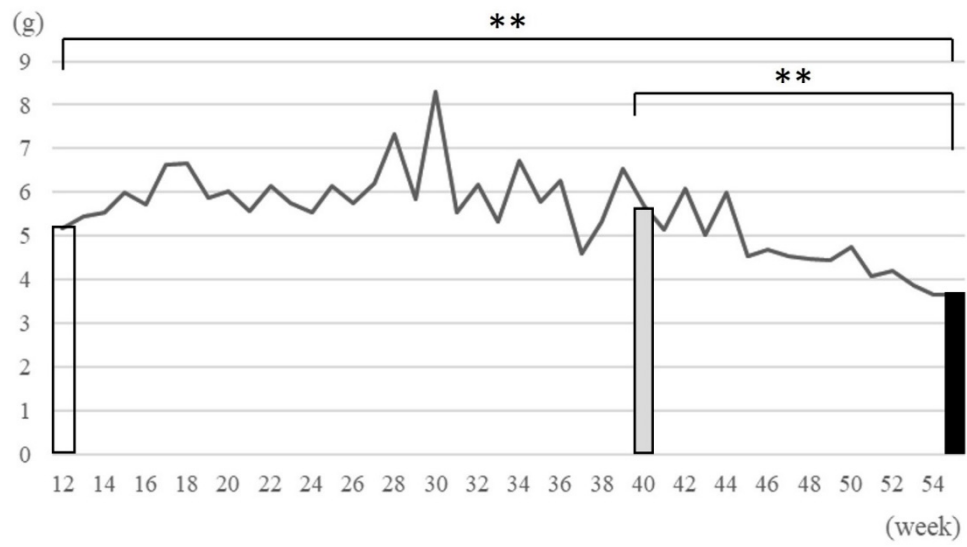

**Figure S1.** The amount of food intake at 55 weeks for five mice over time. \*\*  $p < 0.01$ .

Supplement: Supplementary file 1 [file ijms-25-09684-s001.zip › Supplementary Figure S1.pdf]
